# Pandemic Stressors and Adaptive Responses: A Longitudinal Analysis of the Quality of Life and Psychosocial Dynamics among Urothelial Cancer Patients

**DOI:** 10.3390/jpm13111547

**Published:** 2023-10-28

**Authors:** Vlad Barbos, Bogdan Feciche, Felix Bratosin, Durganjali Tummala, Uday Shree Akkala Shetty, Silviu Latcu, Alexei Croitor, Vlad Dema, Razvan Bardan, Alin Adrian Cumpanas

**Affiliations:** 1 Department XV, Discipline of Urology, Victor Babes University of Medicine and Pharmacy, 300041 Timisoara, Romania; vlad.barbos@umft.ro (V.B.); silviu.latcu@umft.ro (S.L.); alexei.croitor@umft.ro (A.C.); vlad.dema@yahoo.com (V.D.); razvan.bardan@umft.ro (R.B.); cumpanas.alin@umft.ro (A.A.C.); 2 Doctoral School, Victor Babes University of Medicine and Pharmacy, E. Murgu Square, Nr. 2, 300041 Timisoara, Romania; felix.bratosin@umft.ro; 3 Department of Urology, Emergency County Hospital Oradea, Strada Gheorghe Doja 65, 410169 Oradea, Romania; 4 Department XIII, Discipline of Infectious Diseases, Victor Babes University of Medicine and Pharmacy, E. Murgu Square, Nr. 2, 300041 Timisoara, Romania; 5 Department of General Medicine, K.S. Hegde Medical Academy, Mangaluru 575018, India; anju9899@gmail.com; 6 Malla Reddy Institute of Medical Sciences, Suraram Main Road 138, Hyderabad 500055, India; udayshree98@gmail.com

**Keywords:** COVID-19, urothelial cancer, cancer management, quality of life, anxiety

## Abstract

The 2019 coronavirus disease (COVID-19) pandemic has had a profound influence on different sectors of society, including health. This study hypothesized a significant impact of the pandemic on the quality of life and psychosocial well-being of urothelial cancer patients, specifically anticipating a decrease in anxiety and depression scores as the pandemic progressed. The primary objectives were to assess longitudinal changes in quality of life indexes, evaluate Healthcare Anxiety and Depression Scale (HADS) score trends over three years (2020–2022), and identify any correlational patterns between the progression of the pandemic and anxiety, depression, and stress levels among this cohort. A cross-sectional analysis was conducted on Eastern Cooperative Oncology Group (ECOG) 1 and Tumor Node Metastasis (TNM) stage 1 bladder cancer patients from the Timis County Emergency Clinical Hospital in Romania. Sixty patients were evaluated each year from 2020 to 2022, utilizing a detailed selection process involving the review of both the hospital database and paper records. Key data included demographic information, medical history, and responses to the Patient Health Questionnaire (PHQ-9), Short Form (SF-36), HADS, and Generalized Anxiety Disorder (GAD-7) questionnaires. A total of 163 completed questionnaires were analyzed, providing insight into various aspects of patients’ experiences during the pandemic. Notably, the mean hospitalization days ranged from 3.6 ± 2.1 days in 2020 to 4.0 ± 2.4 days in 2022 (*p* = 0.663). Concerns that current symptoms might be pandemic-related spiked to 63.5% in 2021, but reduced to 50.9% in 2022, with this fluctuation being significant (*p* = 0.026). The perception of decreased quality of or accessibility to medical care was significant over the years, with a decline to 52.7% in 2022 (*p* = 0.033). Quality of life assessments demonstrated an upward trend, from an average score of 55.9 ± 8.9 in 2020 to 59.3 ± 8.8 in 2022 (*p* = 0.049). Interestingly, anxiety levels, as indicated by the HADS survey, revealed a significant decline from a score of 7.8 in 2020 to 6.5 in 2022 (*p* = 0.008). On the other hand, GAD-7 scores displayed a downward trend over the years, potentially indicative of developed coping strategies (*p* = 0.034). This study provides a comprehensive insight into the fluctuating dynamics of psychosocial factors and quality of life among urothelial cancer patients during the pandemic years. It underscores a potential adaptive response, as evidenced by the decrease in anxiety levels and an upward trend in the quality of life scores over the period. These findings highlight the resilience and adaptability of this patient cohort amidst the challenges posed by the pandemic, potentially guiding future interventions and supports in similar health crises.

## 1. Introduction

In recent history, the global community has confronted unprecedented challenges emanating from the 2019 coronavirus disease (COVID-19) pandemic, that has brought a cumulative number of 770 million cases and almost 7 million deaths, as of late 2023 [1,2]. The seismic shifts in daily life routines and the healthcare landscape have had profound implications for individuals dealing with chronic illnesses and cancer [3], including those with urological malignancies, marked by its intricate association with various physiological and psychological comorbidities [4,5,6]. Currently, urothelial (bladder) cancer stands as the 10th most common cancer worldwide [7], with a noted increase in incidence, particularly in developed countries where industrial exposures are high [8,9].

Regarding bladder epidemiology in Romania, there are very limited data, although the existing reports suggest an age-standardized incidence of 15.4% [10]. Recent data depict an alarming mortality rate, with an estimated 200,000 deaths annually on a global scale [11]. In terms of survival, the prognosis varies extensively based on the stage at diagnosis; the 5-year disease-free survival rate can exceed 80% for patients diagnosed at an early, localized stage according to the tumor–node–metastasis (TNM stage 1), but drops precipitously to approximately 15% for those with metastatic disease, similarly to other malignancies [12,13,14,15,16]. It is imperative to note that the recurrence rate within 5 years is considerably high, at about 50–70%, necessitating rigorous follow-up strategies post-treatment [17]. Treatment modalities and outcomes are often gauged by the Eastern Cooperative Oncology Group (ECOG) performance status, which aids in tailoring individual therapeutic approaches ranging from surgical interventions to systemic therapies [18,19].

Pre-existing literature has underlined the intricate dynamics between chronic illnesses and various psychological features, further amplified during the times of global crises [20,21]. Thus, delineating the nuanced shifts in the quality of life and psychosocial well-being of urothelial cancer patients during the pandemic stands as a critical question. Cancer patients are known to grapple with a constellation of challenges including, but not limited to, anxiety, depression, and decreased quality of life [22]. Moreover, the COVID-19 pandemic has introduced an additional layer of complexity, imposing unforeseen strains on healthcare systems globally and altering the medical care pathways, potentially exacerbating psychosocial distress among this population [23,24]. Psychological distress, uncertainty and loneliness were described by recent studies as important disturbing factors that people experienced during the COVID-19 pandemic [25,26]. Therefore, a comprehensive investigation into these dynamics during this unique period warrants a detailed exploration to formulate effective interventions and policy adjustments.

The Healthcare Anxiety and Depression Scale (HADS), a renowned instrument in assessing the dual parameters of anxiety and depression, serves as an invaluable tool in quantifying the psychological distress experienced by individuals with cancer [27]. Furthermore, assessing the quality of life, an encompassing metric that captures the overall well-being and life satisfaction of patients, constitutes a critical pillar in the management of cancer, as well as in other critically ill patients [28,29]. The quality of life, often intertwined with physical health, psychological state, level of independence, and personal beliefs, has been notably impacted during the pandemic [30]. In the context of urothelial cancer patients, understanding the multifaceted influences on their quality of life during this period can yield insightful data, potentially driving the development of tailored interventions aimed at fostering resilience and well-being.

In pursuance of a deeper understanding of the impacts of the pandemic on this specific cohort of patients with urothelial cancer of the bladder, this study delineates several key hypotheses and objectives. First, it hypothesizes that the pandemic has exerted a significant influence on the quality of life and psychosocial well-being of urothelial cancer patients. Second, it anticipates lower HADS scores, indicating high levels of anxiety and stress at the beginning of the pandemic, decreasing as the pandemic progresses. To fulfil its primary objectives, this study aims to: (1) investigate longitudinal changes in quality of life indexes, (2) analyze the trends in HADS scores over the three-year period, and (3) identify potential correlational patterns between the pandemic progression and levels of anxiety, depression, and stress among urothelial cancer patients.

## 2. Materials and Methods

### 2.1. Research Design and Ethical Considerations

In line with rigorous academic standards, this current investigation was structured as a descriptive study intending to longitudinally examine data from patients diagnosed with ECOG 1 and TNM stage 1 bladder cancer at the Department of Urology of the Timis County Emergency Clinical Hospital “Pius Brinzeu” in Timisoara, Romania, spanning the years 2020, 2021, and 2022. Adhering to the established ethical protocols, the study was approved by the Local Commission of Ethics for Scientific Research, which operates under provisions aligned with the EU GCP Directives 2005/28/EC, ICH guidelines, and the tenets outlined in the Declaration of Helsinki. The ethical approval for the research was granted approval number 333.

### 2.2. Inclusion Criteria

The selection criteria started with identifying patients with ECOG 1 and TNM stage 1 bladder cancer from the database of the urology clinic within the specified timeline between 2020 and 2022. The initial phase of the selection process involved a meticulous screening of the hospital’s database using the primary diagnosis of urothelial cancer as the keyword, which was further filtered through histopathology results. The selected individuals were adult patients who had exhibited a willingness to contribute personal data for research purposes, as evidenced by the signed consents in their paper records. A diligent review of the paper records facilitated the extraction of pertinent medical details. The exclusion criteria comprised cases with inconsistencies in the database and paper record diagnoses, patients with insufficient data on the considered variables, lack of consent, and incomplete questionnaire results. A total of 60 patients were evaluated every year, while the selected cohort was stratified annually to enable a focused analysis of the variables across the three pandemic years. Patients diagnosed with COVID-19 during the study period were excluded to avoid any confounding variations in the psychometric results. ECOG 1 is defined as restricted in physically strenuous activity, but ambulatory and able to carry out work of a light or sedentary nature, such as light housework or office work [31].

### 2.3. Variables

The study incorporated a comprehensive analysis of variables that extended to patients’ demographics and medical history, alongside specific characteristics pertinent to urothelial cancer and cancer management data [32,33,34]. These encompassed patients’ age, sex, body mass index, history of substance use, referral form, COVID-19 vaccination status, number of comorbidities, tumoral grading, and duration of hospitalization. Additionally, the study also collected information on patients’ responses to the Short Form-36 (SF-36), HADS, Generalized Anxiety Disorder-7 (GAD-7), and the Patient Health Questionnaire-9 (PHQ-9) tools.

### 2.4. Employed Surveys 

The research utilized a suite of validated instruments to determine the various dimensions of the study on the pandemic stressors and adaptive responses in patients with urothelial cancer. The Short Form-36 (SF-36) [35], a globally recognized tool, facilitated the evaluation of the health-related quality of life (HRQOL) and functional status across eight vital domains, including physical functioning, social functioning, and mental health, with scores ranging between 0 and 100 indicating the quality of life. Moreover, the Hospital Anxiety and Depression Scale (HADS) [36] was employed to ascertain the levels of anxiety and depression among the respondents. This 14-item self-report scale splits into two sections—HADS-A and HADS-D—to distinctly assess anxiety and depression levels, providing a comprehensive overview of the psychological state of the patients. To further enhance the depth of the investigation, the study integrated the Generalized Anxiety Disorder-7 (GAD-7) [37] and the Patient Health Questionnaire-9 (PHQ-9) [38] tools to evaluate generalized anxiety disorder symptoms and depression severities, respectively. Besides the four integrated standardized questionnaires, a 10-question unstandardized survey was conducted to assess particularities associated with the COVID-19 pandemic. All patients received the surveys online in the first week after discharge.

### 2.5. Statistical Analysis

Data management and analysis were conducted utilizing the statistical software SPSS version 26.0 (SPSS Inc., Chicago, IL, USA). The sample size was calculated based on a convenience sampling method, with a minimum of 120 respondents, at a 95% confidence level and 10% margin of error, based on the previously reported incidence of approximately 15% of bladder cancer in the Romanian population. Continuous variables were represented as the mean ± standard deviation (SD), while categorical variables were expressed in terms of frequencies and percentages. To analyze the changes between more than two means of continuous variables, an ANOVA test was utilized. The Chi-square test was utilized for the categorical variables. A *p*-value threshold of less than 0.05 was set for statistical significance. All the results were double-checked to ensure accuracy and reliability.

## 3. Results

At the end of the study period, a total of 163 completed questionnaires were collected from the eligible participants, out of which 56 were from 2020, 52 from 2021, and 55 from 2022. The mean age of the participants remained relatively stable throughout the years, being 61.8 ± 9.9 years in 2020, increasing marginally to 62.3 ± 9.5 years in 2021, and slightly decreasing to 60.5 ± 10.2 years in 2022; however, the differences were not statistically significant (*p* = 0.618). The gender distribution across the three years indicated a majority of male participants, constituting approximately 55.4% in 2020, 53.8% in 2021, and increasing to 60.0% in 2022, albeit without a significant difference (*p* = 0.797), as described in Table 1.

Upon analyzing the body mass index (BMI) of participants, it was observed that the mean BMI underwent a slight upward trend, from 26.4 ± 3.9 kg/m^2^ in 2020 to 26.9 ± 4.1 kg/m^2^ in 2021, followed by a dip to 25.8 ± 4.3 kg/m^2^ in 2022, with the variations not attaining statistical significance (*p* = 0.379). Substance use behaviors remained fairly consistent over the years, with chronic smoking being reported in 32.1%, 34.6%, and 27.3% of participants across the respective years (*p* = 0.705), and chronic alcohol use recorded in 8.9%, 5.8%, and 10.9% (*p* = 0.634), respectively.

A majority of the participants originated from urban areas, constituting 62.5% in 2020, 57.7% in 2021, and notably increasing to 70.9% in 2022, though these changes were not statistically significant (*p* = 0.352). Regarding the referral sources, a majority were from secondary care throughout the study period, comprising about 69.6%, 63.5%, and 70.9% in each year, respectively, with no significant difference in the referral patterns (*p* = 0.678). A notable finding was the increase in COVID-19 vaccination rates among the participants, where no vaccinations were reported in 2020, followed by a 23.1% vaccination rate in 2021, and a significant jump to 50.9% in 2022, showcasing a statistically significant increase (*p* = 0.003). When assessing the comorbidities, it was discerned that most participants had up to two comorbidities throughout the three years, and there was no substantial change in the distribution of the number of comorbidities (*p* > 0.05). Nevertheless, the majority of patients were married or in a relationship, although without significant differences regarding relationship status between the three studies years.

Due to the selection process, before surveying the patients, all participants were classified as having TNM stage 1 disease, with an ECOG performance status of 1, indicating that they were fully ambulatory and capable of carrying out work of a light or sedentary nature. Furthermore, all individuals underwent a transurethral resection of the bladder tumor (TURBT), a standard procedure in managing stage 1 bladder cancer. In the context of tumor grading, a slight fluctuation in the distribution of low- and high-grade tumors was noted over the three years. In 2020, 37.5% of patients were diagnosed with low-grade tumors, a fraction that decreased slightly to 34.6% in 2021, before undergoing a rise to 41.8% in 2022. Conversely, high-grade tumors constituted 33.9% in 2020, increasing to 38.5% in 2021, and subsequently decreased to 27.3% in 2022. Furthermore, there was a consistent proportion of cases where the tumor grade remained unknown, oscillating around 28–31% over the years. However, these variations in tumor grading did not attain statistical significance, as indicated by a *p*-value of 0.818, as presented in Table 2.

Additionally, an assessment of the mean hospitalization days revealed a slight upward trend across the three years. Patients hospitalized in 2020 had an average stay of 3.6 ± 2.1 days, which increased marginally to 3.8 ± 2.5 days in 2021, and further to 4.0 ± 2.4 days in 2022. However, this incremental elevation did not translate to a significant difference statistically, with a *p*-value of 0.663, denoting that the differences could be due to random variation.

In terms of experiencing changes in the frequency or severity of urinary symptoms since the onset of the pandemic, there was a gradual increase in the percentage of affirmative responses, from 28.6% in 2020, through 36.5% in 2021, to 38.2% in 2022. However, this increasing trend was not statistically significant, as evidenced by a *p*-value of 0.523. The belief that current symptoms might be associated with the COVID-19 pandemic increased markedly from 37.5% in 2020 to 63.5% in 2021, followed by a decrease to 50.9% in 2022, and was statistically significant with a *p*-value of 0.026. Similarly, changes in access to or quality of medical care were reported consistently by over 70% of respondents in the first two years, with a significant decline to 52.7% reporting changes in 2022, a trend that was found to be significant (*p* = 0.033).

Furthermore, the survey addressed adherence to treatment regimens during the pandemic. The data showed a fluctuation in the responses, with 33.9% affirming challenges in 2020, increasing to 46.2% in 2021, and then decreasing to 27.3% in 2022; this trend, however, did not achieve statistical significance (*p* = 0.118). Similarly, alterations in emotional well-being were noted, though the changes over the years did not amount to statistical significance (*p* = 0.183). In assessing the impact of the pandemic on physical activities or maintaining a healthy lifestyle, the participants rated their experiences on a scale of 1 to 10. A significant downward trend was observed in the scores, moving from 7.3 ± 2.6 in 2020, to 6.5 ± 2.9 in 2021, and then to 6.0 ± 2.4 in 2022 (*p* = 0.034). Meanwhile, the reported levels of stress and anxiety experienced during the pandemic remained relatively stable across the three years, with no significant difference in scores (*p* = 0.466).

When asked to rate their overall quality of life since the onset of the pandemic compared to before, there was a significant upward trend in scores from 2020 to 2022 (*p* = 0.049). Despite these fluctuations, the belief that the pandemic would influence their cancer prognosis and treatment outcomes did not significantly change over the three years (*p* = 0.213). Lastly, changes in social support during the pandemic were noted, with a significant variation in responses over the three years (*p* = 0.019), as presented in Table 3.

Upon observing the physical component of the SF-36 survey, there appeared to be an increasing trend in scores over the years. In 2020, the mean score was 53.0 ± 7.7, which significantly increased to 56.6 ± 7.5 in 2021, and slightly reduced to 55.7 ± 8.0 in 2022. The increment in the scores from 2020 to 2021 and a slight decrease in 2022 indicates a potential variation in the physical health status of urothelial cancer patients during the pandemic years, which was statistically significant, with a *p*-value of 0.043.

Similarly, the mental component of the survey demonstrated an upward trend across the years. The scores exhibited a gradual rise from 51.0 ± 8.1 in 2020 to 53.2 ± 8.4 in 2021, and further to 54.9 ± 8.6 in 2022. This progression suggests a potential improvement in the mental health status of the respondents throughout the pandemic, which was corroborated by a *p*-value of 0.049, denoting statistical significance.

**Figure 1:**
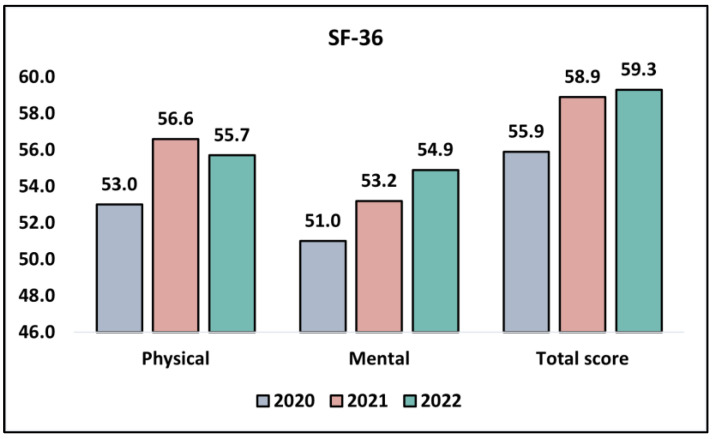
Analysis of the SF-36 questionnaire results during the COVID-19 pandemic.

Lastly, the analysis of the total score from the SF-36 survey indicated an upward trajectory in the perceived health status and quality of life over the three years. The mean score exhibited a climb from 55.9 ± 8.9 in 2020 to 58.9 ± 8.0 in 2021, with a slight further increase to 59.3 ± 8.8 in 2022. Although this trend suggests a possible enhancement in the overall quality of life throughout the pandemic years, this change was not statistically significant, as seen in Table 4 and Figure 1.

When examining the anxiety domain of the HADS survey, we noticed a statistically significant decline in the scores across the three years. In 2020, the mean score was 7.8, which significantly reduced to 6.3 in 2021, and slightly further to 6.5 in 2022. This continuous decrease in the scores signifies a decrease in the levels of anxiety experienced by the participants over the pandemic years, validated by a *p*-value of 0.008, denoting a statistically significant trend. When it comes to the depression domain of the HADS survey, a downward trend in the scores across the years was observed. In 2020, the mean score stood at 6.6, which decreased to 6.0 in 2021 and then to 5.8 in 2022. However, this decline was not statistically significant (*p* = 0.201).

**Figure 2:**
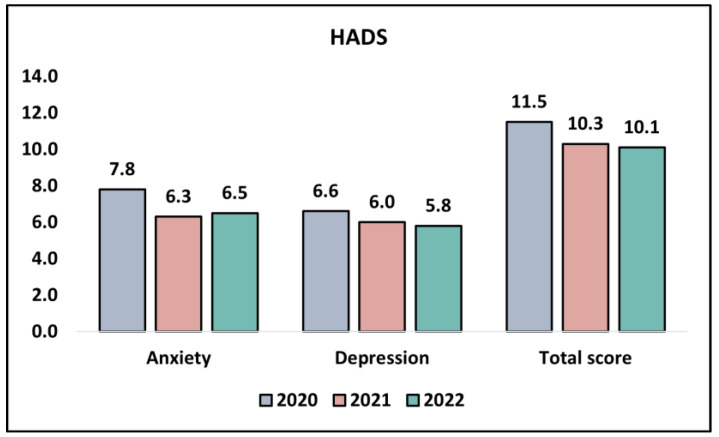
Analysis of the HADS questionnaire results during the COVID-19 pandemic.

Lastly, analyzing the total score from the HADS survey (the aggregate of both the anxiety and depression scores), a gradual decrease was evident from 11.5 in 2020 to 10.3 in 2021, with a further slight reduction to 10.8 in 2022. This trend potentially suggests a decrease in the overall levels of distress among the study participants during the pandemic years. Nevertheless, this change was not statistically significant, with a *p*-value of 0.541, as presented in Table 5 and Figure 2.

**Figure 3:**
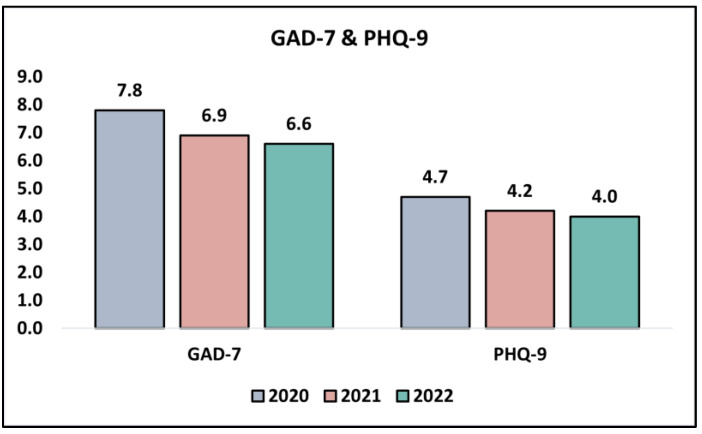
Analysis of the GAD-7 and PHQ-9 questionnaire results during the COVID-19 pandemic.

A scrutiny of the GAD-7 survey results, which is designed to identify potential cases of generalized anxiety disorder, presents a discernible downward trend over the years of the study. In 2020, the participants registered a mean score of 7.8 ± 2.5, which decreased to 6.9 ± 2.2 in 2021 and further to 6.6 ± 2.8 in 2022 (*p* = 0.034). This could potentially signify that the participants experienced a diminution in anxiety symptoms, possibly reflecting adaptive responses or coping strategies that were developed over the period of the pandemic. Turning to the PHQ-9 survey results, which assess the severity of depression symptoms, a marginal decrease in mean scores is observed from 4.7 ± 2.2 in 2020 to 4.2 ± 2.6 in 2021, further decreasing to 4.0 ± 2.3 in 2022. However, this decreasing trend is not statistically significant (*p* = 0.276), as presented in Table 6 and Figure 3.

## 4. Discussion

### 4.1. Important Findings and Literature Review

The present study longitudinally investigated the psychosocial dynamics and quality of life among urothelial cancer patients during the tumultuous period of the COVID-19 pandemic. The analysis encompassed diverse aspects including physical and mental health trends, the influence of the pandemic on their cancer prognosis perception, and shifts in social support structures over a span of three years (2020 to 2022). While some factors exhibited statistically significant shifts, others remained relatively stable. This multi-faceted approach paints a nuanced picture of the complexities that urothelial cancer patients navigated during the pandemic.

The perceived overall quality of life experienced a statistically significant upward trend from 2020 to 2022. Concurrently, there was a noticeable improvement in both the physical and mental components of the SF-36 survey. This perhaps indicates an adaptive resilience developed by the patients during this crisis period, an outcome that mirrors previous findings where individuals facing chronic illnesses exhibit heightened adaptive responses in face of adversity, thereby maintaining or even enhancing their quality of life [39,40,41]. Nevertheless, these patients might have felt less stressed about the pandemic after mass vaccination campaigns and foreseeing the end of the COVID-19 crisis.

Intriguingly, the participants displayed a significant decline in anxiety levels, as evidenced by the GAD-7 and HADS survey results, although the trend in diminishing depression symptoms as per the PHQ-9 and HADS surveys did not attain statistical significance. These observations might point toward the end of the pandemic social restrictions and initiation of a community support system. Earlier research has frequently noted the positive effects of support networks in alleviating anxiety symptoms, especially in cancer patient populations [42,43].

In other larger studies that involved cancer patients impacted by the COVID-19 pandemic, the findings demonstrated prevalence rates of depression at around 25%, anxiety at approximately 20% of the entire cohort, and post-traumatic stress disorder (PTSD) at almost 10% [44]; however, our study did not evaluate the presence of PTSD. Other research revealed considerable psychological distress in hemato-oncology patients, with a notable percentage grappling with anxiety (36%), depression (31%), and PTSD (36%), especially noticeable among young women [45]. In contrast, our study uncovered no notable differences in the levels of depression evaluated by the HADS and GAD-7. Also, the current study did not include patients who were previously infected with the SARS-CoV-2 virus, while it was found in different research that individuals who had been directly affected by COVID-19 exhibited more signs of psychological trauma and depression [46].

The prevalence of depression in cancer patients, as evidenced in the literature, varied widely, with rates documented between 0 and 40% [47]. Several potential triggers for anxiety, depression, and distress were identified in cancer patients, including factors like low self-esteem, limited social support, and decreased functional abilities, recognized as risk factors for depression [48]. Nevertheless, it is imperative to highlight the widespread occurrence of PTSD symptoms in cancer patients during the pandemic, with cancer-related PTSD rates reaching up to 30% [49]. This higher-than-expected rate could be attributed to fears of COVID-19 infection and increased mortality risks, illustrating the extensive psychological distress and PTSD symptoms that accompany trauma exposure, especially in individuals who have encountered death [50].

In line with our observations, other studies also noted significant alterations in bladder cancer management in the year following the pandemic. Although there has been a gradual decrease in the occurrence of urothelial cancer over the past two decades, the drastic reduction observed in other studies surpasses the estimated annual decline of around 1% [51]. This could potentially be attributed to the prolonged periods of reduced elective operations witnessed in numerous hospitals post COVID-19 onset. Interestingly, there was not a substantial increase in the waiting times for urothelial cancer surgeries during the respective periods, suggesting a diminished incidence of urothelial cancer since the advent of the pandemic. Nevertheless, a recent review emphasized the adverse consequences of delays between the diagnosis of bladder cancer and subsequent surgical interventions on overall survival outcomes, accentuating the necessity for prompt surgical management in treating urothelial cancers [52].

The current study captured a significant increase in COVID-19 vaccination rates over the years, an encouraging trend highlighting the intensified immunization efforts. However, fluctuations were noted in the perceptions of social support, indicating the potential restructuring or strain in social networks during the pandemic. Previous literature has emphasized the vital role that consistent social support plays in enhancing the quality of life among cancer patients; thus, these variations warrant deeper investigations to facilitate better psychosocial support strategies [53].

### 4.2. Study Limitations and Future Perspectives

In discussing the limitations of the current study, it is essential to acknowledge the cross-sectional design of this research, which primarily restricts our ability to draw causal relationships between the variables studied and the outcomes observed. While the study meticulously stratified the cohort annually, it investigated the pandemic stressors and adaptive responses across a relatively short time frame of three years, which might not fully encapsulate the long-term effects of the pandemic on the quality of life and psychosocial dynamics among patients with urothelial cancer. Also, the small sample size increases the risk of a type 1 error. Moreover, the study relied heavily on self-reported data from the survey instruments, potentially introducing response biases that might affect the reliability of the findings. Furthermore, the specific focus on patients with ECOG 1 and TNM stage 1 bladder cancer, while allowing for a more homogeneous study group, might limit the generalizability of the findings to broader populations with varying stages and types of urothelial cancer. Lastly, although extensive, the set of variables considered might not encompass all the potential factors that could influence the psychosocial dynamics investigated.

## 5. Conclusions

Considering the current results, it is evident that the pandemic has impacted a series of significant alterations in the psychosocial well-being and perceived quality of life among patients with urothelial bladder cancer. These patients exhibited higher stress and anxiety levels at the onset of the pandemic, associated with a decreased quality of life and HADS. A noteworthy finding from this study is the demonstrable resilience displayed by this group, as illustrated by the decreasing trends in anxiety levels and a simultaneous improvement in the quality of life scores across the years 2020 to 2022. This may suggest the evolution of coping mechanisms and adaptability in the face of prolonged health crises, but it can also be caused by the introduction of vaccination campaigns that decreased the infection risks and continued toward ending the pandemic. However, the observed fluctuations in concerns regarding the potential pandemic-related nature of their symptoms and the perceived decline in the quality of or accessibility to healthcare emphasize the necessity for continuous, adaptive support systems within healthcare settings. Future studies should seek to further substantiate these findings and delineate the specific factors that facilitated these adaptive responses to foster resilience and better preparedness in managing patient well-being during subsequent global health emergencies.

## Figures and Tables

**Table 1:** Background characteristics of patients with urothelial cancer by the year of diagnosis and intervention.

	2020 (*n* = 56)	2021 (*n* = 52)	2022 (*n* = 55)	*p*-Value *
Age, years (mean ± SD) **	61.8 ± 9.9	62.3 ± 9.5	60.5 ± 10.2	0.618
Sex (male)	31 (55.4%)	28 (53.8%)	33 (60.0%)	0.797
BMI, kg/m^2^ (mean ± SD) **	26.4 ± 3.9	26.9 ± 4.1	25.8 ± 4.3	0.379
Substance use behavior				
Chronic smoking	18 (32.1%)	18 (34.6%)	15 (27.3%)	0.705
Chronic alcohol use	5 (8.9%)	3 (5.8%)	6 (10.9%)	0.634
Place of origin (urban)	35 (62.5%)	30 (57.7%)	39 (70.9%)	0.352
Referral Source				0.678
Primary care	17 (30.4%)	19 (36.5%)	16 (29.1%)	
Secondary care	39 (69.6%)	33 (63.5%)	39 (70.9%)	
COVID-19-vaccinated	-	12 (23.1%)	28 (50.9%)	0.003
Number of comorbidities				
0–1	14 (25.0%)	10 (19.2%)	16 (29.1%)	
2	30 (53.6%)	29 (55.8%)	25 (45.5%)	
≥3	12 (21.4%)	13 (25.0%)	14 (25.5%)	
Relationship status				0.679
Single/divorced/widowed	7 (12.5%)	10 (19.2%)	9 (16.4%)	
In a relationship/married	40 (71.4%)	34 (65.4%)	41 (74.5%)	
Unknown	9 (16.1%)	8 (15.4%)	5 (9.1%)	
Surveying period				0.312
1st quarter	7 (12.5%)	9 (17.3%)	11 (20.0%)	
2nd quarter	20 (35.7%)	15 (28.8%)	12 (21.8%)	
3rd quarter	17 (30.4%)	21 (40.4%)	16 (29.1%)	
4th quarter	12 (21.4%)	7 (13.5%)	16 (29.1%)	

* Chi-square or Fisher’s exact test; ** ANOVA test; SD—standard deviation; BMI—body mass index.

**Table 2:** Oncological features of patients with urothelial cancer by the year of diagnosis and intervention.

	2020 (*n* = 56)	2021 (*n* = 52)	2022 (*n* = 55)	*p*-Value *
Tumoral infiltration (NMIBC)	0 (0.0%)	0 (0.0%)	0 (0.0%)	-
TNM stage 1	56 (100%)	52 (100%)	55 (100%)	-
ECOG 1	56 (100%)	52 (100%)	55 (100%)	-
TURBT	56 (100%)	52 (100%)	55 (100%)	-
Grading				0.818
Low grade	21 (37.5%)	18 (34.9%)	23 (41.8%)	
High grade	19 (33.9%)	20 (38.5%)	15 (27.3%)	
Unknown	16 (28.6%)	14 (26.9%)	17 (30.9%)	
Days of hospitalization (mean ± SD) **	3.6 ± 2.1	3.8 ± 2.5	4.0 ± 2.4	0.663

* Chi-square or Fisher’s exact test; ** ANOVA test; NMBIC—non-muscle-invasive bladder cancer; SD—standard deviation; ECOG—Eastern Cooperative Oncology Group; TURBT—transurethral resection of bladder tumor.

**Table 3:** Unstandardized survey results.

Question	2020 (*n* = 56)	2021 (*n* = 52)	2022 (*n* = 55)	*p*-Value *
Since the onset of the pandemic, have you experienced any changes in the frequency or severity of your urinary symptoms? (Yes, %)	16 (28.6%)	19 (36.5%)	21 (38.2%)	0.523
Do you believe your current symptoms might be associated with the COVID-19 pandemic? (Yes, %)	21 (37.5%)	33 (63.5%)	28 (50.9%)	0.026
During the pandemic, have there been any changes in your access to medical care or the quality of the medical care you received? (Yes, %)	41 (73.2%)	38 (73.1%)	29 (52.7%)	0.033
Did you feel challenged in adhering to any treatment regimens during the pandemic? (Yes, %)	19 (33.9%)	24 (46.2%)	15 (27.3%)	0.118
Have you noticed any changes in your emotional well-being? (Yes, %)	37 (66.1%)	30 (57.7%)	41 (74.5%)	0.183
Has the pandemic affected your ability to engage in physical activities or maintain a healthy lifestyle? (rate on a 1 to 10 scale) **	7.3 ± 2.6	6.5 ± 2.9	6.0 ± 2.4	0.034
How often did you experience stress and anxiety symptoms during the pandemic? (rate on a 1 to 10 scale) **	6.9 ± 3.1	6.2 ± 3.0	6.6 ± 2.7	0.466
How would you rate your overall quality of life since the onset of the pandemic compared to before? **	5.1 ± 2.2	5.8 ± 2.4	6.4 ± 3.5	0.049
Do you believe the pandemic will influence your cancer prognosis and treatment outcomes? (Yes, %)	24 (42.9%)	20 (38.5%)	15 (27.3%)	0.213
Have you experienced any changes in social support during the pandemic? (Yes, %)	29 (51.8%)	33 (63.5%)	20 (36.4%)	0.019

* Chi-square or Fisher’s exact test; ** ANOVA test.

**Table 4:** SF-36 survey results stratified by the COVID-19 pandemic years.

SF-36 (Mean ± SD)	2020 (*n* = 56)	2021 (*n* = 52)	2022 (*n* = 55)	*p*-Value
Physical	53.0 ± 7.7	56.6 ± 7.5	55.7 ± 8.0	0.043
Mental	51.0 ± 8.1	53.2 ± 8.4	54.9 ± 8.6	0.049
Total score	55.9 ± 8.9	58.9 ± 8.0	59.3 ± 8.8	0.077

SD—standard deviation; SF-36—Short Form Survey (higher scores indicate a better health status and quality of life).

**Table 5:** HADS survey results stratified by the COVID-19 pandemic years.

HADS (Mean ± SD)	2020 (*n* = 56)	2021 (*n* = 52)	2022 (*n* = 55)	*p*-Value
Anxiety	7.8 ± 2.9	6.3 ± 3.0	6.5 ± 2.2	0.008
Depression	6.6 ± 2.1	6.0 ± 2.8	5.8 ± 2.4	0.201
Total score	11.5 ± 5.3	10.3 ± 5.0	10.1 ± 5.8	0.541

SD—standard deviation; HADS—Hospital Anxiety and Depression Scale (higher scores indicate levels of anxiety or depression).

**Table 6:** GAD-7 and PHQ-9 survey results stratified by the COVID-19 pandemic years.

Variables (Mean ± SD)	2020 (*n* = 56)	2021 (*n* = 52)	2022 (*n* = 55)	*p*-Value
GAD-7	7.8 ± 2.5	6.9 ± 2.2	6.6 ± 2.8	0.034
PHQ-9	4.7 ± 2.2	4.2 ± 2.6	4.0 ± 2.3	0.276

SD—standard deviation; GAD—General Anxiety Disorder (higher scores indicate higher anxiety symptoms); PHQ—Patient Health Questionnaire (higher scores indicate more severe depression symptoms).

## Data Availability

The data presented in this study are available on request from the corresponding author.
